# Mycoplasma-induced minimally conscious state

**DOI:** 10.1186/s40064-016-1832-2

**Published:** 2016-02-24

**Authors:** Thomas Horvath, Urs Fischer, Lionel Müller, Sebastian Ott, Claudio L. Bassetti, Roland Wiest, Parham Sendi, Joerg C. Schefold

**Affiliations:** Department of Neurology, Inselspital, Bern University Hospital, 3010 Bern, Switzerland; Department of Intensive Care Medicine, Inselspital, Bern University Hospital, 3010 Bern, Switzerland; Department of Pulmonary Medicine, Inselspital, Bern University Hospital, 3010 Bern, Switzerland; Department of Diagnostic and Interventional Neuroradiology, Bern University Hospital, 3010 Bern, Switzerland; Department of Infectious Diseases, Inselspital, Bern University Hospital, 3010 Bern, Switzerland

**Keywords:** Encephalitis, Myelitis, ICU, Coma, *Mycoplasma pneumoniae*, Atypical pneumonia

## Abstract

*Mycoplasma pneumoniae* (*M. pneumoniae*) frequently causes community-acquired respiratory tract infection and often presents as atypical pneumonia. Following airborne infection and a long incubation period, affected patients mostly suffer from mild or even asymptomatic and self-limiting disease. In particular in school-aged children, *M. pneumoniae* is associated with a wide range of extrapulmonary manifestations including central nervous system (CNS) disease. In contrast to children, severe CNS manifestations are rarely observed in adults. We report a case of a 37 year-old previously healthy immunocompetent adult with fulminant *M. pneumoniae*-induced progressive encephalomyelitis who was initially able to walk to the emergency department. A few hours later, she required controlled mechanical ventilation for ascending transverse spinal cord syndrome, including complete lower extremity paraplegia. Severe *M. pneumoniae*-induced encephalomyelitis was postulated, and antimicrobial, anti-inflammatory and immunosuppressive therapy was applied on the intensive care unit. Despite early and targeted therapy using four different immunosuppressive strategies, clinical success was limited. In our patient, locked-in syndrome developed followed by persistent minimally conscious state. The neurological status was unchanged until day 230 of follow-up. Our case underlines that severe *M. pneumoniae*- related encephalomyelitis must not only be considered in children, but also in adults. Moreover, it can be fulminant and fatal in adults. Our case enhances the debate for an optimal antimicrobial agent with activity beyond the blood–brain barrier. Furthermore, it may underline the difficulty in clinical decision making regarding early antimicrobial treatment in *M. pneumoniae* disease, which is commonly self-limited.

## Background

*Mycoplasma pneumoniae* (*M. pneumoniae*) is a small bacterium that lacks a rigid cellular wall. It is considered highly contagious and airborne infection may result in atypical pneumonia following a longer incubation period (Waites and Talkington 2004; Waites and Atkinson 2009). Most often, *M. pneumoniae*-induced atypical pneumonia can be treated in an outpatient setting and the clinical course is commonly mild and self-limiting or even asymptomatic (Spuesens 2014). Importantly, in particular in school-aged children (Christie et al. 2007), *M. pneumoniae* is associated with a wide range of extrapulmonary manifestations including mucocutaneous and central nervous system (CNS) disease. In few cases severe encephalomyelitis develops (Waites and Talkington 2004; Christie et al. 2007; Daxboeck 2006; Granerod et al. 2010; Tsiodras et al. 2005; Atkinson and Waites 2014). Here we report a rare case of *M. pneumoniae*-infection with resulting locked-in syndrome progressing to minimally conscious state in a previously healthy immunocompetent adult.

## Case history

A 37-year-old Caucasian woman presented to the emergency department with a 10-day history of productive cough, low-grade fever (38.5 °C), generalized myalgia, and lower extremity weakness. Several days before onset of symptoms, her 2-year old daughter experienced mild dyspnea and fever. At clinical examination, basal lung field crepitation was noted. The WBC count was 13 g/L and C-reactive protein level 38 mg/L. Neurological examination revealed homonymous left hemianopsia, reduced sensitivity to tactile/painful stimuli (below Th6-level), lower extremity paraparesis (global strength M2–M3) with areflexia, and urinary retention. Strength of upper extremities was tested normal. Except for bilateral interstitial pulmonary infiltrates, whole body CT-scans returned unremarkable. A cerebral CT angiogram and a subsequent MR-angiogram did not reveal signs of vasculopathy.

Within 2 h, complete lower extremity paraplegia and ascending transverse spinal cord syndrome (Th2-level) developed and required invasive controlled mechanical ventilation. Cerebral and spinal MRI showed extensive myelitis. Differential diagnoses of fulminant encephalomyelitis included infectious, parainfectious, autoimmune/inflammatory, paraneoplastic, and acquired CNS demyelinating disease. Lumbar puncture revealed polynucleated pleocytosis (95 M/L WBC, 67 % polynucleated; protein 3.34 g/L, lactate 6.90 mmol/L, glucose 2.67 mmol/L) with positively tested polyclonal bands. In addition to clarithromycin, the empirical antimicrobial treatment consisted of ceftriaxone, ampicillin, and acyclovir. CSF-analyses for HSV-I/-II, VZV, CMV, EBV, enteroviruses, tuberculosis, bacterial growth, and serological testing for HIV, syphilis, tick-borne encephalitis, hepatitis, and autoimmune disease (anti-nuclear-, anti-neutrophil cytoplasmatic-, anti-cardiolipin-, and neuromyelitis optica antibodies) were negative. However, nasopharyngeal swab (PCR), serum antibodies, and CSF-tests returned positive for *M. pneumoniae* [serum-IgG 159 U/L (normal range <20 U/L); serum-IgM 46 U/L (<13 U/L)], with a positive serum to CSF IgM-ratio [3.8 (<1.3)]. The diagnosis of rapidly progressive *M. pneumoniae*-induced encephalomyelitis was postulated (Tsiodras et al. 2005). Few hours following initial admission, the antimicrobial treatment was streamlined to moxifloxacin (400 mg daily), tetracycline, and pulse methylprednisolone (1 g/day) was added thereafter for 7 days (Carpenter 2002).

**Fig. 1:**
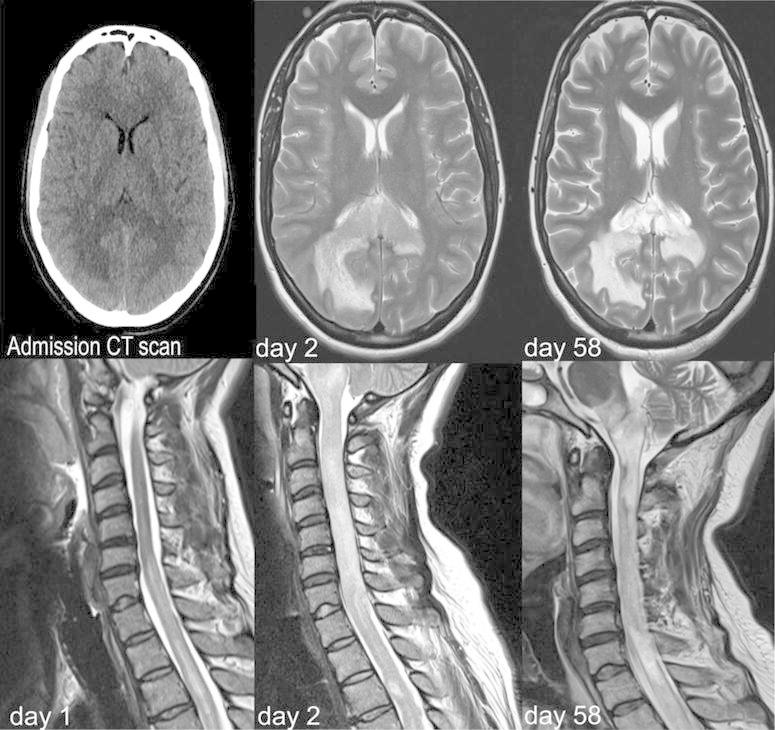
Baseline cerebral CT scan (*upper left side*) and cerebral and spinal MRI at days 1, 2, and 58, respectively

On day 2, flaccid quadriplegia with complete areflexia and sensory loss (C3-level) developed. Communication was possible by means of head movement only. Finally, cranial nerve function diminished with only vertical eye movement preserved and locked-in syndrome was diagnosed. Repeated MRI demonstrated a diffusion restriction accompanied by predominantly subcortical T2-hyperintensities (along splenium/corpus callosum, inferior parietal lobe, brainstem) extending into the cervical spine (Fig. 1). Supported only by anecdotal data (Cotter et al. 1983), plasmapheresis for severe para-infectious encephalomyelitis was performed (five sessions QD, followed by five sessions QOD). With decreasing alertness, cyclophosphamide (1 g/dose, days 10 and 24) and infliximab (375 mg/m^2^, day 40) was administered without clinical effect. A corpus callosum biopsy revealed extensive demyelination, though PCR for *M. pneumoniae* was negative. A tracheostomy for long-term mechanical ventilation was performed on day 10 and moxifloxacin was continued for a total of 21 days. Prednisolone was progressively tapered and in the further course and the daily dosage at discharge was 25 mg.

**Fig. 2:**
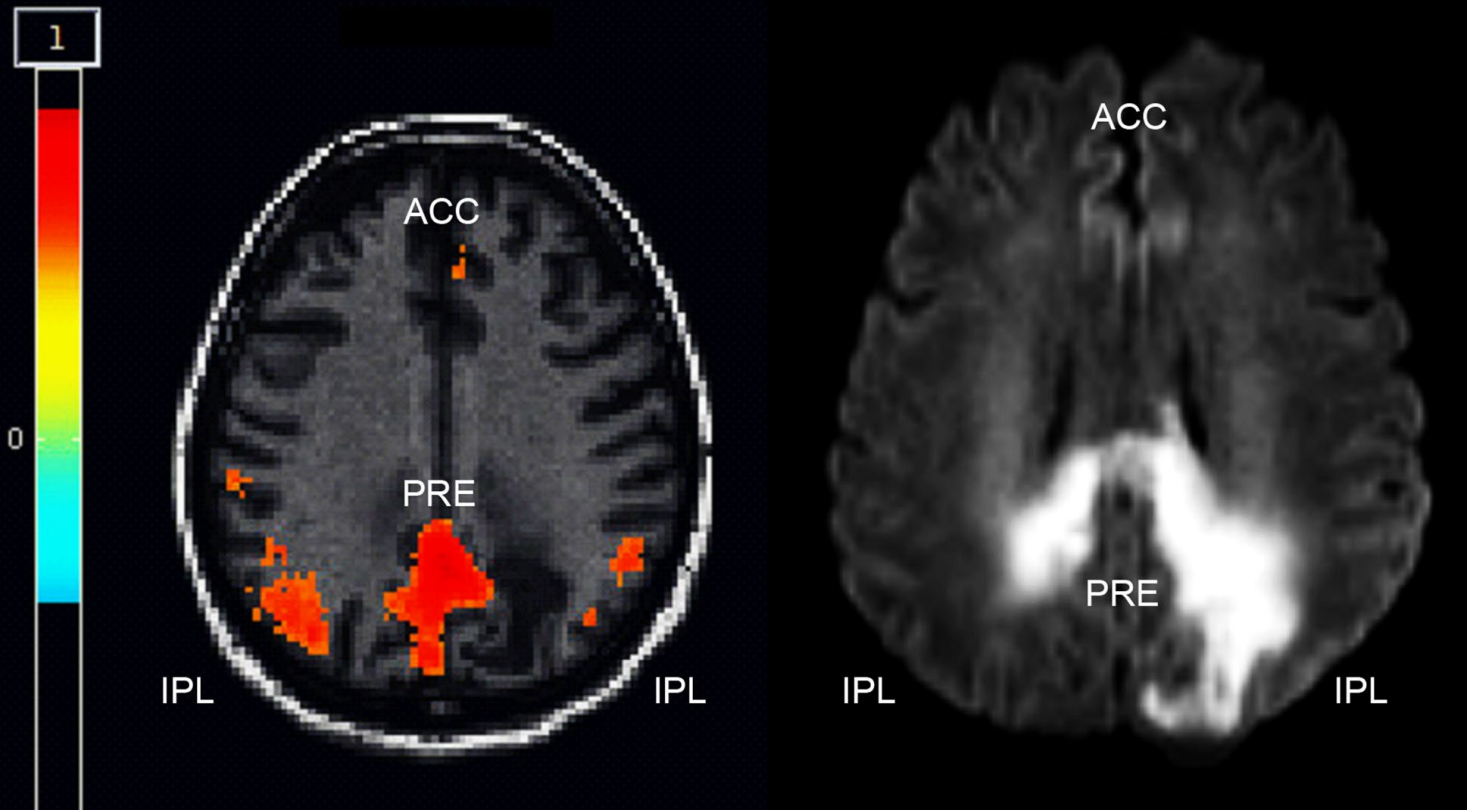
Resting state functional MRI (with central seed placed in the precuneus, PRE). *IPL* and *ACC* denote inferior parietal lobe and anterior cingulate cortex, respectively. Images in neurological convention; *red areas* indicate connectivity between PRE and IPC/ACC >0.5 (Whitfield-Gabrieli and Nieto-Castanon 2012). *Left* multiband EPI-sequence (TR 300 ms, TE 30 ms, 32 contiguous slices, matrix size 64 × 64, FoV 1380 mm); *right* diffusion weighted imaging sequence (b = 1000, TE 3500 ms, TE 89 ms, matrix size 128 × 128, FoV 230 mm)

At day 58, eye movements lapsed and MRIs showed extensive necrotizing encephalitis with hemorrhagic transformation and blood–brain barrier disruption within cervicospinal lesions (Fig. 1). Resting-state functional MRI to assess default mode network (DMN) connectivity was performed (Fig. 2) (Whitfield-Gabrieli and Nieto-Castanon 2012). Connectivity was preserved in the posterior DMN i.e. between the precuneus (a core structure regarding connectivity loss in comatose patients) and inferior parietal lobe, and a reduction between posterior DMN and anterior cingulate cortex. Thus, clinical and imaging findings indicated progression of locked-in syndrome to minimally conscious state (Demertzi et al. 2015). Finally, following extensive communication with next-of-kin, the patient was discharged to a nursing home on controlled mechanical ventilation. Follow-up until day 230 confirmed a persistent neurological status.

## Discussion

The case presented here demonstrates fulminant progression of transverse encephalomyelitis. Main differential diagnoses include systemic autoimmune disease, paraneoplastic and acquired CNS demyelinating disease (such as neuromyelitis optica or multiple sclerosis), as well as infectious, postinfectious, or paravaccinal disorders, and acute demyelinating encephalomyelitis (Frohman and Wingerchuk 2010).

Today, underlying pathomechanisms for *M. pneumonia*e-induced encephalomyelitis remain incompletely understood. Currently debated major pathomechanisms include direct bacteremia-induced or other invasive (i.e. *per continuitatem*) CNS-injury, effects related to bacterial neurotoxins, as well as immune-complex mediated or molecular mimicry-induced (auto-)immune responses (Daxboeck 2006; Tsiodras et al. 2005; Spuesens et al. 2014). Although the consequences are currently unclear, e.g. polyclonal B cell activation in combination with production of distinct auto-antibodies were previously described in *M. pneumoniae* disease (Biberfeld 1971). Nevertheless, evidence on optimal treatment for severe *M. pneumoniae*-induced encephalomyelitis is limited. This clinical dilemma seems further aggravated by the fact that diagnosis of *M. pneumoniae* induced meningo-encephalitis by serological tests seems challenging due to lack of assays with high sensitivity and specificity. Nevertheless, before the background of the assumed immunopathogenesis of the disease, most experts recommend immunosuppressive therapy (Daxboeck 2006; Tsiodras et al. 2005).

In the case presented here, we applied early anti-microbial therapy with postulated sufficient CSF-blood ratio and four different immunomodulating strategies right after onset of acute neurological symptoms.

Importantly, current available data do not allow concluding on optimal choice, timing and dosage of immunomodulating strategies in this clinical setting and the role of additional infection-induced acquired immunodeficiency (Hotchkiss et al. 2013; Schefold et al. 2008) remains enigmatic. However, despite advanced immunosuppressive treatment, it seems worth to note that severe secondary infection was not noted in our patient. Unfortunately, we observed that despite early anti-microbial, anti-inflammatory and immunosuppressive therapy, clinical success was limited in our patient.

The clinical course of our patient underlines that *M. pneumoniae*-induced encephalomyelitis is a severe condition that must not only be considered in younger children but also in adults. In addition, this case underlines the difficulty in clinical decision making regarding early antimicrobial treatment in *M. pneumoniae* disease, which is commonly self-limited.
